# Men with Sickle Cell Anemia and Priapism Exhibit Increased Hemolytic Rate, Decreased Red Blood Cell Deformability and Increased Red Blood Cell Aggregate Strength

**DOI:** 10.1371/journal.pone.0154866

**Published:** 2016-05-04

**Authors:** Kizzy-Clara Cita, Laurent Brureau, Nathalie Lemonne, Marie Billaud, Philippe Connes, Séverine Ferdinand, Benoit Tressières, Vanessa Tarer, Maryse Etienne-Julan, Pascal Blanchet, Jacques Elion, Marc Romana

**Affiliations:** 1 Inserm UMR 1134, Université des Antilles, Pointe à Pitre, Guadeloupe; 2 Laboratoire d’Excellence du Globule Rouge (LABEX GR-Ex), COMUE Sorbonne Paris Cité, Paris, France; 3 CHU de Pointe-à-Pitre, Service d’Urologie, Pointe-à-Pitre, Guadeloupe; 4 Inserm, U1085—IRSET, Pointe-à-Pitre, Guadeloupe, France; 5 CHU de Pointe-à-Pitre, Unité Transversale de la Drépanocytose, Centre de référence maladies rares de la drépanocytose aux Antilles-Guyane, Pointe-à-Pitre, Guadeloupe; 6 Institut Universitaire de France, Paris, France; 7 Université Claude Bernard Lyon 1, COMUE Lyon, Laboratoire LIBM EA 7424, Team “Vascular Biology and red blood cell”, Lyon, France; 8 Inserm, CHU de Pointe-à-Pitre, Centre d’Investigation Clinique Antilles Guyane CIC 14–24, Guadeloupe, France; 9 Inserm U 1134, Paris, France; Emory University/Georgia Insititute of Technology, UNITED STATES

## Abstract

**Objectives:**

To investigate the association between priapism in men with sickle cell anemia (SCA) and hemorheological and hemolytical parameters.

**Materials and Methods:**

Fifty-eight men with SCA (median age: 38 years) were included; 28 who had experienced priapism at least once during their life (priapism group) and 30 who never experienced this complication (control group). Twenty-two patients were treated with hydroxycarbamide, 11 in each group. All patients were at steady state at the time of inclusion. Hematological and biochemical parameters were obtained through routine procedures. The Laser-assisted Optical Rotational Cell Analyzer was used to measure red blood cell (RBC) deformability at 30 Pa (ektacytometry) and RBC aggregation properties (laser backscatter *versus* time). Blood viscosity was measured at a shear rate of 225 s^-1^ using a cone/plate viscometer. A principal component analysis was performed on 4 hemolytic markers (i.e., lactate dehydrogenase (LDH), aspartate aminotransferase (ASAT), total bilirubin (BIL) levels and reticulocyte (RET) percentage) to calculate a hemolytic index.

**Results:**

Compared to the control group, patients with priapism exhibited higher ASAT (*p* = 0.01), LDH (*p* = 0.03), RET (*p* = 0.03) levels and hemolytic indices (*p* = 0.02). Higher RBC aggregates strength (*p* = 0.01) and lower RBC deformability (*p* = 0.005) were observed in patients with priapism compared to controls. After removing the hydroxycarbamide-treated patients, RBC deformability (*p* = 0.01) and RBC aggregate strength (*p* = 0.03) were still different between the two groups, and patients with priapism exhibited significantly higher hemolytic indices (*p* = 0.01) than controls.

**Conclusion:**

Our results confirm that priapism in SCA is associated with higher hemolytic rates and show for the first time that this complication is also associated with higher RBC aggregate strength and lower RBC deformability.

## Introduction

Sickle cell anemia (SCA), one of the most common hemoglobinopathies worldwide, is a multisystem disorder with diverse clinical manifestations and a wide inter-individual variability in its clinical presentation [1]. SCA is caused by a point mutation in the β-globin gene resulting in the substitution of glutamic acid by valine at position 6 of the β-globin chain (Glu6Val). The resulting abnormal hemoglobin S (HbS) polymerizes when deoxygenated, causing a mechanical distortion of the red blood cells (RBC). The deformed short living sickle-shaped RBCs can interact with endothelial cells, leukocytes, platelets and other plasma components to initiate the typical vaso-occlusive manifestations of the disease. During the past decade, new insights into SCA pathophysiology have been described through assessment of the intravascular hemolysis consequences on nitric oxide (NO) bioavailability. This has led to the enticing, but still debated, proposal of two main SCA phenotypes which may explain the associated acute and chronic complications: the hemolytic-endothelial dysfunction and viscosity-vaso-occlusion phenotypes [2, 3].

Priapism, defined as an either painful or painless, purposeless and persistent state of penile erection in the absence of, or abnormally following, sexual stimulus [4], is a frequent SCA manifestation in adult men, with a reported prevalence of 30% to 45% [5–7]. Priapism can occur as brief, repetitive clusters known as stuttering episodes or as a major event (acute priapism) which cause penile tissue ischemia and inflammatory reaction and may result in penile fibrosis, erectile dysfunction and, ultimately, impotence [4, 8].

The pathophysiology of SCA-related priapism remains only partially understood. Initially, low-flow (ischemic) priapism, which is the form of this complication typically associated with SCA, was presumed to result from the slugging of sickle RBCs in the cavernosal sinusoids, thereby interfering with venous outflow [9]. Acute cavernosal hypoxia may cause further RBC sickling and, eventually, paralysis of the cavernosal smooth muscle leading to a priapic event [10]. More recently, several studies suggested that alteration in erection physiology regulatory signaling pathway, namely a dysfunction of NO/phosphodiesterase-5 (PDE5) pathway and an impairment of adenosine signaling, could be involved in the occurrence of priapism [11–14]. Despite its prevalence, there is no consensus on the optimal therapeutic intervention for ischemic priapism.

While priapism has also been assigned to the hemolytic-endothelial dysfunction phenotype [2, 15], this point remains controversial [16–18]. Blood flow in both macro- and microcirculation is influenced by blood rheology, such as blood viscosity, RBC deformability and aggregation properties [19]. Impaired blood rheology participates to the occurrence of several acute and chronic complications in SCA [20]. However, the contribution of blood rheology to priapic events has never been studied. In this report, we determine the hemorheological and hematological parameters in men with SCA and compare these parameters between men who had and had not experienced priapism.

## Materials and Methods

### Study Population

This prospective non-interventional study was performed in a single center, at the University Hospital of Pointe-à-Pitre (Guadeloupe, French West Indies). From March 2014 to January 2016, 58 adult men with SCA who were regularly being followed by the Reference Sickle Cell Center of Guadeloupe were included while they were at steady state. Steady state was defined as no blood transfusion in the previous three months and no acute episodes, such as infection, painful vaso-occlusive crises, acute chest syndrome, or stroke (but not priapism), in the previous 2 months. Among the included participants, 22 were already under hydroxycarbamide (HC) treatment with an average dose of 14.7 mg/kg per day. For all patients on HC who also had a past history of priapism, the treatment had been started after the onset of priapism. No consensus on the time duration necessary to define an episode of priapism has been established so far; the average duration of this complication has been reported to be 125 min, ranging from 30 min to 480 min [16]. In this study, priapism was defined as a painful erection lasting more than 1 hour and requiring medical care, similar to in other studies [15, 17]. The definition of pulmonary hypertension, leg ulcer and stroke were defined as previously described [21–23]. SCA diagnosis was previously established in the hemoglobinopathy laboratory of the Pointe-à-Pitre Hospital by isolectrofocusing electrophoresis (Multiphor IITM System, GE HEALTH CARE, Buck, UK), high performance liquid chromatography (VARIANT™, Bio Rad Laboratories, Hercules, CA, USA) and had been confirmed through DNA studies [24]. Polymerase Chain Reaction (Gap-PCR) had been used to detect common alpha-thalassemia deletions [25] [26]. All included patients were informed about the purpose and procedures of the study and gave written consent. The study was conducted in accordance with the guidelines set by the declaration of Helsinki and was approved by the Regional Ethics Committee (CPP Ile de France IV, Paris, France, n°IRB 00003835, registration number: 2012-23NICB).

### Biological parameters

In the course of this study, venipuncture was performed between 8 a.m. and 10 a.m. and EDTA blood samples were immediately used for measurements of hemoglobin concentration (Hb), hematocrit (Hct), mean corpuscular volume (MCV), mean corpuscular hemoglobin (MCH), reticulocytes (RET), red blood cell (RBC), platelets (PLT) and white blood cell (WBC) counts (Max M-Retic, Coulter, USA). Measurements of biochemical parameters (total bilirubin, BIL; lactate dehydrogenase, LDH; aspartate aminotransferase, AST) were performed using standard biochemistry.

### Hemorheological parameters

All hemorheological measurements were carried out within 4 hours of the venipuncture and after complete oxygenation of the blood, according to the internationally standardized guidelines for blood rheology techniques and measurements [27]. Blood viscosity was measured at native hematocrit (Brookfield DVII+ cone-plate viscometer, CPE40-spindle, Middelboro, MA, USA), 25°C and 225 s^-1^. RBC deformability was determined at 30 Pa and 37°C by ektacytometry (LORCA, RR Mechatronics, Hoorn, The Netherlands). The recent methodological recommendations for RBC deformability measurement in SCA by ektacytometry were strictly followed [28]. RBC aggregation (RBC aggregation index) was determined at 37°C by syllectometry (i.e., laser backscatter *versus* time) and after adjustment of the hematocrit to 40% [27]. Hematocrit standardization was done by removing the adequate volume of plasma after blood centrifugation. The RBC disaggregation threshold, i.e., the minimal shear rate needed to prevent RBC aggregation or to breakdown pre-existing RBC aggregates (RBC aggregate strength), was determined using a re-iteration procedure [29].

### Statistical analysis

According to the Shapiro-Wilk test for normality, the quantitative variables were summarized as means ± standard deviation or as medians with the interquartile range (IQR) while qualitative variables were expressed as percentages. A data reduction technique called principal component analysis (PCA), was used to create a summary variable (hemolytic index) that would capture most of the information of the original data using four hemolytic parameters (BIL, LDH, ASAT and RET), as has been done previously [30]. PCA is a standard statistical approach which is proven to be useful for studying underlying mechanisms reflected in an individual’s biological measurements [31]. The hemolytic index derived from PCA is a normalized factor of the four hemolytic variables with a mean of 0 and explains 59.2% of the total variance of the four variables. Differences between patient groups were assessed using the unpaired Student’s t-test and Mann-Whitney U test for continuous covariates or the chi-square test for categorical covariates. Pearson’s correlation test was used to test the presence of correlation. The significance level was defined as *p <* 0.05. Analyses were conducted using SPSS (v. 21, IBM SPSS Statistics, Chicago, IL, USA).

## Results

Among the 58 men with SCA (median age: 39 years, IQR: 26–46) included in this study, 28 had experienced priapism at least once during their life and are hereafter referred to as the priapism group. The time between their inclusion in the study and their last onset of priapism was: more than 2 months (n = 18), one month (n = 5) and less than 1 week (n = 5).

The frequency of priapism events was variable from a patient to another. Three patients had experienced only single event during their life while 23 patients reported several priapic episodes punctuated by long time periods without priapism. For two patients, an estimated frequency of priapic events was not available. Among the other 23 men with several events, 10 experienced an average of less than one episode per year. The average priapism frequency for the other 13 patients in this group ranged as followed: one episode per year (n = 3), one episode per month (n = 5), one episode per week (n = 4) and 1 patient was experiencing approximately one priapic event per day at the time of the study. The median age of the first priapism episode was 17 years (range: 9 to 36 years). Most of these patients (n = 21) described stuttering priapism, which refers to transient, painful, and repetitive episodes lasting less than 3 hours, treated mostly at home by self-intrapenile injection of etilefrine, and warm baths or showers. Three patients had at least one major episode (lasting more than 4 hours) treated by surgery at the University hospital of Guadeloupe. Six other episodes were also treated in the University hospital; 4 by intrapenile injection of etilefrine and 2 by blood transfusion.

Table 1 summarizes the hematological and hemorheological parameters of both the priapism and control groups. The median age and proportion of patients in each group who were under HC treatment were similar. We detected no significant differences in Hb and HbF levels, MCV values, WBC and PLT counts and α-thalassemia frequency. In contrast, we detected significantly higher levels of ASAT, LDH, RET percentage and hemolytic indices and a trend for bilirubin level in the priapism group compared to the control group.

**Table 1:** Hematological, hemorheological and clinical parameters of men with SCA, according to the occurrence of priapism.

	Priapism	No priapism	*p* values
n	28	30	-
Age (years)	34 (25–43)	40 (28–50)	0.19
HU treatment n (%)	11 (39%)	11 (37%)	1
Hb (g/dL)	8.6 (7.6–9.4)	8.5 (7.5–9.6)	1
MCV (fl)	86.2 ± 13.9	89.7 ± 13.8	0.34
HbF (%)	6.2 (3.6–13.6)	4.7 (2.1–9.0)	0.34
RET (%)	8.2 (6.2–10.5)	6.2 (4.2–9.0)	**0.03**
LDH (IU/L)	442 (381–618)	348 (292–493)	**0.03**
Total BIL (μM)	62 (31–114)	38 (23–62)	0.06^§^
ASAT (IU/L)	34 (31–42)	28 (23–34)	**0.01**
Hemolytic index	0.52 ± 1.7	-0.50 ± 1.2	**0.02**
Alpha-thalassemia n (%)	9 (33.3%)	15 (50%)	0.23
WBC (10^9^/L)	9.0 (6.5–9.6)	7.9 (5.1–9.1)	0.13
PLT (10^9^/L)	331 (262–390)	277 (223–385)	0.26
Hematocrit (%)	25.8 ± 3.3	28.0 ± 5.1	0.1
Blood viscosity (225 s^-1^)	5.0 (4.2–5.5)	4.7 (4.2–5.8)	0.97
RBC deformability (30 Pa)	0.34 (0.26–0.44)	0.44 (0.37–0.51)	**0.005**
RBC aggregation index (%)	51.4 (43.1–54.0)	51.8 (48.3–57.7)	0.39
RBC disaggregation threshold (s^-1^)	300 (200–575)	213 (160–344)	**0.01**
Leg ulcer n (%)	10 (35.7%)	9 (30%)	0.78
Pulmonary Hypertension n (%)	2 (7.1%)	0 (0%)	0.23
Stroke n (%)	1 (3.6%)	0 (0%)	0.48

Quantitative variables were summarized as median with the interquartile range (IQR) or as means ± standard deviation. Qualitative variables are described by number and percent. Intergroup differences were assessed using Mann Whitney test, chi-square test, or unpaired Student’s t-test as appropriate. Significant *p* values are in bold.

^§^indicates a trend (*p*<0.1).

In terms of hemorheological parameters, blood viscosity and RBC aggregation indices were not different between the two groups. However, we detected lower RBC deformability and higher RBC disaggregation thresholds in the priapism group compared to the control group. In addition, an inverse relationship was detected between the hemolytic index and RBC deformability (r = -0.58, *p* < 10^−5^) and between RBC deformability and the RBC disaggregation threshold (r = -0.46, *p* = 0.0004).

No differences were detected between the priapism group and the control group for the occurrence of other complications such as leg ulcers, pulmonary hypertension and stroke.

HC treatment is known to modulate both hematological and hemorheological parameters [32–34] and to be associated with a variable inter-individual response [35, 36]. By comparing hematological parameters of patients without priapism before and during HC treatment, we detected a significant rise of MCV (81.3 ± 5.7 *vs* 99.6fl ± 11.8, *p* = 0.0002), a modest increase of HbF (4.1 ± 2.4 *vs* 6.9% ± 3.2, *p* = 0.039) and a trend for higher Hb level (7.9 ± 1.2 *vs* 8.4g/dl ± 1.7, *p* = 0.09) following HC treatment. In contrast, HC treatment in the priapism group seemed to lead to a substantial increase of not only MCV (81.4 ± 4.9 *vs* 101fl ± 11.6, *p* = 0.0002) but also HbF expression (3.5 ± 1.35 *vs* 12.3% ±5.3, *p* = 0.0004) and Hb level (7.1 ± 1.2 *vs* 8.8g/dl ± 1.1, *p* = 0.0005). Altogether, these data suggested slightly better HC-treatment responders in the priapism group. Therefore, we re-did the statistical analyses after removing the SCA patients treated with HC. Once patients on HC treatment were removed from analysis, the priapism group exhibited lower hematocrit (28.4 ± 4.9 *vs* 25.5% ± 3.1, *p* = 0.04) and RBC deformability (0.45 [0.31–0.52] *vs* 0.30Pa [0.26–0.39], *p* = 0.01) and higher RBC disaggregation threshold (190 [148–340] *vs* 325s^-1^ [256–375], *p* = 0.03) than the control group. The priapism group also exhibited higher RET percentage (8.9 [6.4–13.4] *vs* 6.3% [4.7–9.4], *p* = 0.03), BIL level (91 [43–157] *vs* 48μM [25–75], *p* = 0.04), LDH rate (476 [392–683] *vs* 366IU/L [288–465], *p* = 0.001) and hemolytic indices (1.14 ± 1.76 *vs* -0.29 ± 1.30, *p* = 0.01) as well as a trend for higher ASAT (36 [30–43] *vs* 27IU/L [23–34], *p* = 0.07) when compared to the control group.

## Discussion

In this study, we show that men with SCA who have a positive history of priapism have higher hemolytic rates, lower RBC deformability, higher RBC aggregate strength (i.e., higher RBC disaggregation thresholds) and, to a lesser extent, lower hematocrit when compared to men with SCA who never experienced priapism.

Several conflicting studies have been reported on the hematological characteristics associated with the occurrence of priapism in males with sickle cell disease (SCD). The largest study published as of yet was the Comprehensive Study of Sickle Cell Disease which included 1252 men with SCD, of whom 296 were affected by sickle cell–hemoglobin C disease [15]. In this study, a history of priapism was found to be associated to low Hb levels, elevated LDH and bilirubin levels and reticulocyte percentages while the patient was at steady-state. However, the same research group, in a subsequent cohort study of 677 men with SCA, did not detect any association between priapism and low Hb values [17]. A survey conducted in children and adolescents with SCA and sickle cell/ β^0^-thalassemia found no relationship between a positive history of priapism and baseline hematological data [16]. Similar results to this were obtained in a cohort of 123 adolescents and men with SCA [18]. These discrepancies remain puzzling and could be related to differences in various factors such as, study settings, sickle cell genotype of the patients analyzed, patient ages and effects of treatment (ex. hydroxycarbamide), all of which are known to modify the hematological profile of patients with SCA [32, 33].

Our results demonstrated higher hemolytic rate in men with SCA men who had a positive history of priapism compared to those who had never experienced this complication. After the removal of patients treated with hydroxycarbamide in the two groups, a lower Hct accompanied higher hemolytic rates in the priapism group, but not in the control group. It is also worthwhile to mention that the frequency of α-thalassemia, a condition known to be associated with reduced hemolytic rates [37], is lower, although not significantly, in the priapism group.

Our research group has recently shown that the hemorheological parameters of patients with SCA differs according to the types of complications considered [20]. For example, patients with SCA who also exhibited high frequency of vaso-occlusive complications have higher RBC deformability [38, 39] and blood viscosity [38, 40, 41] when compared to those with a low rate of vaso-occlusive complications. In contrast, patients with glomerulopathy and leg ulcers, two complications assigned to the hemolytic-endothelial dysfunction phenotype, are characterized by lower RBC deformability and higher RBC aggregates strength than SCA patients without these complications [22, 42].

Similar to glomerulopathy and leg ulcers, priapism is usually considered to be a hemolytic complication [2]. In such case, one could expect that these three complications would share common hemorheological profile, and our findings clearly support this, with priapic SCA patients exhibiting reduced RBC deformability and increased RBC aggregate strength when compared to the control group. Both a reduction in RBC deformability and the persistence of non-disaggregated and sticky RBC aggregates may impair blood flow in macro- and microcirculation [19], which may ultimately alter tissue perfusion [20]. The negative correlation found between RBC deformability and the hemolytic index confirms previous findings showing that RBCs with the highest rigidity are the most fragile (i.e. those more prone to hemolysis) [43]. In addition, increased robustness of RBC aggregates was associated with decreased RBC deformability, as previously demonstrated [43]. Enhanced oxidative stress caused, in part, by increased hemolysis [2] could be the common factor responsible for both increased RBC aggregate strength and decreased RBC deformability [44] in men with SCA who have recurrent priapism; further studies are needed to test this hypothesis.

The high rate of hemolysis which occurs in patients with SCA results in a decrease of NO bioavailability and enhanced oxidative stress, which in turn impairs vascular reactivity [2]. It has been suggested that NO depletion induces a compensatory down regulation of phosphodiesterase type 5 protein expression and activity, leading to an accumulation of cGMP in the corporal smooth muscle and thus rendering the penile vasculature uncontrollably dilated [12]. In this study, we have shown that patients with a positive history of priapism are characterized by the presence of very robust and rigid RBC aggregates, which are likely to flow in vascular networks with limited vasomotor reserve due to impaired NO bioavailability. This hemorheological picture is highly conceivable along with the known pathophysiology of priapism in men with SCA considering that these poorly deformable RBCs create robust aggregates that may favor venous stasis, obstructing the penile vessels and leading to priapic events. Each of the above mentioned abnormalities as well as others, such as dysregulation of adenosine signaling pathway [13, 14], may play a role in the occurrence of priapism in SCA, and further studies are needed to decipher their respective significance in the etiology of this complication.

One of the limits of this study is the relative small number of men with SCA who were included in the analysis. The lack of association between priapism and the other hemolytic complications studied may indeed result from the limited statistical power of our study. However, it should be noted that all of the patients included in this study regularly attended the Sickle Cell Center of Guadeloupe, which reduces the effect of heterogeneous medical follow-up on the clinical results of the study. Another limitation is that most of the patients in the priapism group exhibited stuttering priapism and thus our findings may not be relevant for patients with major priapic attacks. Further studies are warranted to address this issue.

## Conclusion

In summary, our study confirms that priapism in men with SCA is associated with higher hemolytic rates and has shown, for the first time, that this complication is also associated with higher RBC aggregate strength and lower RBC deformability; two hemorheological features which may disturb blood flow in microcirculatory territories.
